# Outcomes of Mini-Percutaneous Nephrolithotomy in Children and Adolescents: A 10-Year Single-Centre Experience From Kuwait

**DOI:** 10.7759/cureus.25022

**Published:** 2022-05-15

**Authors:** Mohamed Zeid, Hani Sayedin, Abdulnaser Alsaid, Natrajan Sridharan, Arun Narayanaswa, Subhasis Giri, Fawzi Abul, Shabir Almousawi

**Affiliations:** 1 Urology, Limerick University Hospital, Limerick, IRL; 2 Urology, Warrington and Halton Teaching Hospitals NHS Foundation Trust, Warrington, GBR; 3 Urology, Ibn Sina Hospital, Kuwait, KWT; 4 Urology, Sabah Al Ahmad Urology Centre, Kuwait, KWT; 5 Urology, University Hospital Limerick, Limerick, IRL

**Keywords:** pediatrics, pediatric urinary stone disease, pcnl complications, mini-percutaneous nephrolithotomy, percutaneous nephrolithotomy (pcnl), renal calculi

## Abstract

The current study retrospectively reviewed data for all children and adolescents who underwent mini-percutaneous nephrolithotomy (PCNL) at Ibn Sina Hospital and Sabah Al Ahmad Urology Centre in Kuwait over 10 years. Accordingly, the 40 patients underwent mini-PCNL. Among them, 21 patients (52.5%) had varying degrees of hydronephrosis, with mild to moderate severity accounting for nearly half of them, whereas six (15%) had multiple stones. The median operative time was 54.5 (43.3-64) minutes. Moreover, 11 patients needed flexible ureteroscopy (URS) and double-J (DJ) ureteric stent, and one patient required DJ ureteric stent only. None of the cases developed intraoperative bleeding. The median hospital stay of the included patients was three (2.3-4) days. Residual stone was observed in 11 patients (27.5%), with a median size of 3 (2 to 7) mm. The incidence of postoperative complications was 27.5% (n = 11 patients), with three patients experiencing postoperative bleeding (7.5%) and eight patients developing a fever (20%). All patients had mild postoperative pain. However, no leakage, sepsis, or pelvic injury occurred. None of the patients required revision. In conclusion, mini-PCNL was a safe and effective procedure in children and adolescents with renal stones.

## Introduction

Paediatric urolithiasis is a serious health problem. As such, efforts to determine the best treatment for such a condition are ongoing. Extracorporeal shock wave lithotripsy (ESWL) has been commonly used as first-line therapy in the United States [1]. However, concerns have been raised regarding the long-term safety of ESWL including slight functional differentiation, measured by the glomerular filtration rate (GFR), of the growing kidney [2]. Even following ESWL, any remaining stones might cause recurrence in children due to the greater prevalence of metabolic and structural abnormalities [3].

Additionally, given that preventing retreatment in children and adolescents remains a major issue, any approach that might lead to a stone-free outcome should not be restricted or excluded [4]. According to the literature, children who receive conventional percutaneous nephrolithotomy (PCNL) have a clearance rate between 50% and 98% [5-7]. The optimal treatment should be minimally invasive to achieve a high stone-free rate (SFR) and reduced retreatment rates [8].

Paediatric and adult patients have been treated successfully using the modified standard PCNL known as minimally invasive PCNL (mini-PCNL) [9,10]. Compared to adults, children have a weaker pelvicalyceal system and lower tolerance to blood loss, resulting in a more difficult situation for urologists [11]. Previous studies have characterized the outcomes of paediatric PCNL using various surgical instruments and age groups [12-14]. According to a recent systematic review, the use of mini-PCNL in children and adolescents has been demonstrated to be both safe and effective [15]. The present study was conducted to determine the various features and outcomes of paediatric and adolescent patients who had undergone mini-PCNL at a single centre in Kuwait.

## Materials and methods

This study was approved by the Institutional Review Board (IRB) committee of Sabah Al Ahmad Urology Centre in Kuwait. The need for informed consent was waived as per hospital policy. We affirm that all study procedures comply with the Declaration of Helsinki principles [16]. The STROBE guidelines were followed during the drafting of this manuscript [17].

Study design and patients

The present two-centre retrospective chart review study was conducted at Sabah Al Ahmad Urology Centre and Ibn Sina Hospital, which has a urology department being a part of Sabah Al Ahmed Urology Centre, in Kuwait for over 10 years (from 2009 to 2019). Data from children and adolescents (aged less than 18 years old) who underwent mini-PCNL at our centre were retrieved. All patients were required to have normal kidney function to be included. Patients with no postoperative follow-up data, renal anomalies, solitary kidney, or renal transplants were excluded.

Data collection and statistical analysis

The following data were retrieved from the medical records of eligible patients: age, sex, anthropometric measurements, complaints, renal calculus characteristics, preoperative renal function values, preoperative haemoglobin levels, urine culture findings, surgical characteristics and duration, intra and postoperative complications, hospital stay, number of residual stones, and need for a second operation. At our centre, all mini-PCNL procedures were performed under general anaesthesia, using a 5-6 Fr ureteric catheter in a retrograde fashion. The access track is usually dilated using a 15 or 16 Fr Amplatz sheath.

Data were analysed using the SPSS version 0.25 software for Windows (IBM Corp, Armonk, NY). We used frequencies to summarize categorical data, whereas continuous data were presented as means ± standard deviation (SD).

## Results

Over a 10-year period, 40 patients underwent mini-PCNL with a median age of seven (interquartile range [IQR] 4.1-9) with male predominance (62.5%). Nearly two-thirds of the patients presented with pain. Only one patient had a history of neurological problems. The median weight and height of the patients were 24.5 (IQR 16.9-28.9) kg and 120 (IQR 105-130) cm, respectively. Most of the patients had unilateral stones (62.5%), mainly on the right side, whereas 21 patients (52.5%) had varying degrees of hydronephrosis, with nearly half of them having mild to moderate severity (n = 11 patients). The median preoperative serum creatinine and haemoglobin levels were 44 (40-55) mg/dL and 14 (13-14) g/dL, respectively. None of the patients showed growth in their urine culture. Six patients (15%) had multiple stones. The median stone length and width were 16 (13-18) and 3.5 (3-4) cm, respectively. The median Hounsfield units (HU) was 775 (680-850). Six patients (15%) had a history of recurrent stones (Table 1).

**Table 1:** Preoperative data of the study group (n=40) ^*^IQR: Interquartile range; **DM: Diabetes Mellitus; ***HTN: Hypertension; ****EWSL: Extracorporeal shock wave lithotripsy; *****HU: Hounsfield density

Variables		Patients (n=40)	
		N	%
Age (years)	Median (IQR)*	7 (4.1 – 9)	
Sex	Males	25	62.5
Comorbidities	DM**	4	6.7
	HTN***	4	6.7
Presentation	Failure EWSL****	7	17.5
	Haematuria on and off	6	15.0
	Pain	26	65.0
	Recurrent infection	1	2.5
History	Neurological problem	1	2.5
Weight (Kg)	Median (IQR)	24.5 (16.9 – 28.9)	
Height (cm)	Median (IQR)	120 (105 – 130)	
Side	Bilateral	15	37.5
	Left	7	17.5
	Right	18	45.0
Hydronephrosis	Mild	3	7.5
	Mild to Moderate	11	27.5
	Moderate	5	12.5
	No hydronephrosis	19	47.5
	Severe hydronephrosis	2	5.0
Serum creatinine (mg/dL)	Median (IQR)	44 (40 – 55)	
Hemoglobin (g/dL)	Median (IQR)	14 (13 – 14)	
Stone Burden	Multiple stone	6	15.0
	Partial staghorn	15	37.5
	Staghorn stone	19	47.5
Stone length in cm	Median (IQR)	16 (13 – 18)	
Stone Width in cm	Median (IQR)	3.5 (3 – 4)	
HU*****	Median (IQR)	775 (680 - 850)	
Recurrent stone		6	15.0

All patients underwent min-PCNL in the prone position. Seven patients (17.5%) required two puncture trials. The Amplatz sheath was 15 Fr in 92.5% of the cases. The median operative time was 54.5 (43.3-64) min. A DJ stent in one patient and a flexible ureteroscopy and DJ in 11 patients were needed. Moreover, none of the cases developed intraoperative bleeding. All patients showed intraoperative clearance under the image intensifier (Table 2).

**Table 2:** Intraoperative data of the study group (n=40) *DJ: Double J; **URS: Ureteroscopy

Variables		Study group (n=40)	
		N	%
Operation time (min)	Median (IQR)	54.5 (43.3 – 64)	
Puncture	Single	33	82.5
	Two	7	17.5
Stone extraction	Laser with Lithoclast	10	25.0
	Laser	30	75.0
Clearance at operation		40	0
Bleeding		0	0
DJ* tube		1	2.5
Flexible URS** and DJ		11	27.5

The median postoperative serum creatinine and haemoglobin levels were 49 (45-55.3) mg/dL and 12.9 (12.6-13.8) g/dL, respectively. Patients had a median hospital stay of 3 (2.3-4) days. Eleven patients (27.5%) had residual stones, with a median size of 3 (2-7) mm. The incidence of postoperative complications was 27.5% (n = 11 patients), with three patients having postoperative bleeding (7.5%) and eight having fever (20%). All patients had mild postoperative pain, with no incidence of leakage, sepsis, or pelvic injury. None of the patients required revision (Table 3).

**Table 3:** Postoperative data of the study group (n=40)

Variables		Study group (n=40)	
		N	%
Laboratory findings	Serum creatinine (mg/dL)	49 (45 – 55.3)	
	Hemoglobin (g/dL)	12.9 (12.6 – 13.8)	
Hospital stay (days)		1.40	0.62
Residual stone	0	29	72.5
	1	7	17.5
	2	4	10.0
Residual stone size (mm)	Median (IQR)	3 (2 -7)	
Postoperative pain	Mild	40	100
Bleeding		3	7.5
Post-operative leakage		0	0
Postoperative fever		8	20.0
Post-operative sepsis		0	0
Postoperative pelvic injury		0	0

## Discussion

All patients in the current retrospective study underwent min-PCNL in the prone position, with 17.5% requiring two puncture trials. Moreover, 92.5% of the cases used a 15 Fr Amplatz sheath. The median operative time was 54.5 minutes, whereas the median hospital stay was three (2.3-4) days. In addition, no intraoperative bleeding was noted, with all patients exhibiting intraoperative clearance under the KUB. Approximately 27.5% of the cases had residual stone, with a median size of 3 (2-7) mm. The incidence of postoperative complications was 27.5%, including postoperative bleeding and fever. All patients had mild postoperative pain, with no incidence of leakage, sepsis, or pelvic injury. Moreover, none of the patients required revision.

An Iraqi study investigating the role of mini-PCNL in children with complex staghorn stones reported an SFR of 78%, with 17% of the patient developing serious complications. These findings are consistent with the results of our study and previous studies, which showed an SFR and complication rate of 58%-94% and 13%-42%, respectively [18-22]. Despite the higher complication rates in our study, the severity of the complications ranged from mild to moderate. Some investigators have suggested that the overall complication rate was significantly correlated to procedure time, the number of access tracts, and stone size and complexity [23,24]. Blood transfusions have been most concerning in paediatrics, with an estimated incidence of 24% [25]. Controversy has arisen regarding the use of adult-sized instruments (F24-30) over mini-PCNL among juvenile patients. Certain studies have shown that decreasing the instrument diameter did not affect complication rates [23], whereas others have found the opposite [26,27]. Evidence in adults has shown that reducing the scope and tract diameter was beneficial for minimizing bleeding complications, both intra- and postoperative [27].

A systematic review of eight studies showed that the pooled mean stone size was 1.2 cm (range: 0.8-3.5 cm), and the most prevalent location of stone was the lower pole and renal pelvis (57% and 24.3%). The pooled mean operative time and length of hospital stay were 76.8 min (range: 20-120 minutes) and 4.6 days (range: 1-33 days), respectively. The pooled overall SFR was 97%. Conventional PCNL was not required in any of the cases. Among the individuals who underwent the procedure, 19% experienced complications. The mean transfusion rate reported across the studies was 3.3% [15].

Zeren et al., who utilized nephrostomy tracts ranging from 18 to 30 Fr for paediatric PCNL, reported an SFR of 87%, postoperative fever rate of 30%, and transfusion rate of 24% [28]. Another study found higher transfusion rates in children with nephrostomy tracts larger than 20 Fr, although transfusion with a 14 Fr was not needed [29]. Guven et al. performed PCNL with only one nephrostomy tract among infants with complicated renal stones. They found a significant reduction in haemoglobin levels when the tract was larger than 20 Fr [26]. Interestingly, it was found that a 24 Fr tract in an infant is comparable to a 72 Fr tract in adults [30]. As such, children's nephrostomy catheters should be between 14 and 20 Fr in diameter.

A single-centre study conducted by Brodie et al. on 46 patients aged between 1 and 16 years reported a complete stone clearance rate of 76%, with no intra or postoperative blood transfusion or mortality. The Amplatz sheath size used was 16 Fr or less. In addition, Yan et al. found that Mini-PCNL monotherapy (tract size 14-16 Fr) cleared 85.2% of stones, with no children requiring blood transfusions [31]. Zeng et al. described their experience with children in 331 renal units, showing a stone clearance rate of 80.4% and blood transfusion rate of 3.1%. Owing to significant bleeding, two children had bladder washouts, another two had pleural injuries that necessitated an intercostal chest drain, and one developed an abscess around the kidney after surgery [32].

The current study found median postoperative haemoglobin of 12.9 (12.6-13.8) g/dL. Studies by Ozden et al. [33], Desai et al. [13], and Manohar et al. [34] have shown that haemoglobin decreases by an average of 1.6, 1.9, and 2.2 g/dL, respectively, in paediatric patients undergoing PCNL for difficult calyceal and staghorn calculi. They observed that the decrease in haemoglobin was linked to the number and size of the nephrostomy tracts. Patients with several nephrostomy tracts experienced a statistically significant increase in blood loss and transfusions.

The operative time obtained in our study was much lower than that reported by Zeng et al., who reported a mean operative time of 73.6 ± 20.2 minutes in children. However, they noted that children and adolescents had a shorter operative time compared to adults [32]. This may be explained by the results of Falahatkar et al., who found a significant correlation between operative time and the number of nephrostomy tracts, amount of stone burden, and position of the calyx for access (upper > lower > middle calyx) [35].

## Conclusions

PCNL is a common surgical procedure in adults; however, this is not the situation in the paediatric age group. There are limited modalities to treat urolithiasis in children. From our experience, mini-PCNL might be safe and effective for children with renal stones. In the majority of the patients, stone clearance with few complications may be achieved using a single nephrostomy tract. Selecting the optimal nephrostomy tract diameter should be investigated in managing this group of patients.
